# Assessment of Guideline-Directed Medical Therapy Optimization Scores and Readmission Risk in Heart Failure With Reduced Ejection Fraction

**DOI:** 10.1177/10600280251387249

**Published:** 2025-11-14

**Authors:** Hanna Jensen, Max Staskauskas, Sara Strome, Jethro Pobre, Nghi Le, Cameryn Nakamura, Mina Fahmy, Benjamin Dang, Yvette Hellier, Jaekyu Shin, Tiffany K. Pon, Trina Huynh, Jennifer Namba, Louise T. Wang, Christine Cadiz, Zaid Yousif, Andrew Willeford

**Affiliations:** 1 Department of Pharmacy, UC San Diego Health, San Diego, CA, USA; 2 UC San Francisco Health, San Francisco, CA, USA; 3 UC Davis Medical Center, Sacramento, CA, USA; 4 Ronald Reagan UCLA Medical Center, Los Angeles, CA, USA; 5 San Francisco School of Pharmacy, University of California, San Francisco, CA, USA; 6 Skaggs School of Pharmacy and Pharmaceutical Sciences, University of California San Diego, La Jolla, CA, USA; 7 Irvine School of Pharmacy and Pharmaceutical Sciences, University of California, Irvine, CA, USA

**Keywords:** heart failure, hospital readmission, guideline-directed medical therapy, score

## Abstract

**Background:**

Heart failure with reduced ejection fraction carries high morbidity and mortality. Guideline-directed medical therapy (GDMT) improves outcomes, yet real-world use is often suboptimal. Multiple scoring tools quantifying GDMT optimization have been developed to identify areas of improvement, including GDMT count, Optimal Medical Therapy (OMT), modified OMT (mOMT), and Kansas City Medical Optimization (KCMO) scores. Their clinical utility in predicting readmissions is uncertain.

**Objective:**

To evaluate the association between four GDMT scoring systems and 30- and 90-day heart failure (HF) readmissions.

**Methods:**

This multi-center, retrospective cohort study included adults with a left-ventricular ejection fraction ≤40% hospitalized for HF across five academic medical centers between February 2021 and July 2023. Patients discharged on ≥1 GDMT class with ≥1 healthcare encounter within 90 days were included. GDMT scores were calculated at discharge, and their associations with 30- and 90-day HF readmissions were analyzed using mixed-effects Cox proportional hazards models adjusted for clinical covariates.

**Results:**

Among 544 patients, 13.1% experienced 30-day and 26.8% experienced 90-day HF readmissions. At 30 days, no GDMT score was significantly associated with readmission. At 90 days, higher OMT (hazard ratios [HR]: 0.93, 95% CI: 0.86-0.99) and mOMT (HR: 0.94, 95% CI: 0.88-0.99) scores were associated with lower readmission risk, whereas GDMT count and KCMO scores were not. Patients on minimal GDMT regimens contributed disproportionately to 90-day readmissions.

**Conclusion and Relevance:**

In this multi-center cohort, OMT and mOMT scores, but not GDMT count or KCMO, were associated with 90-day HF readmissions. Findings highlight the potential utility of simpler GDMT scoring systems while underscoring the need for refined tools incorporating dose intensity, class-specific weighting, and longitudinal therapy to better guide optimization efforts.

## Background

Heart failure (HF) affects more than 64 million people worldwide and is associated with significant economic burden.^1^ In patients with HF with reduced ejection fraction (HFrEF), clinical guidelines strongly recommend initiation and optimization of four classes of guideline-directed medical therapies (GDMT) including (1) angiotensin-neprilysin receptor inhibitors (ARNI)/angiotensin converting enzyme inhibitors (ACEi)/angiotensin-II receptor blockers (ARBs), (2) evidence-based beta blockers (BB) (i.e., bisoprolol, carvedilol, metoprolol succinate), (3) mineralocorticoid receptor antagonists (MRA), and (4) sodium-glucose cotransporter 2 inhibitors (SGLT2i) to reduce morbidity and mortality.^2^ Despite robust evidence supporting the use of these agents in HFrEF, real-world use of GDMT in patients who are eligible for therapy remains suboptimal.^3^

Considering the frequent suboptimal use of GDMT, multiple scoring tools have been developed to quantify the optimization of a patient’s GDMT regimen and identify areas of improvement. Published tools include GDMT count, Optimal Medical Therapy (OMT) score, modified Optimal Medical Therapy (mOMT) score, the Kansas City Medical Optimization (KCMO) score, and a computable algorithm that integrates a medication optimization score (MOS). The GDMT count uses an integer system on a scale from 0 to 4 to measure the presence of the 4 pillars of GDMT where more classes is associated with improved clinical outcomes, including reduced risk of cardiovascular death and HF hospitalization.^4^ The OMT score uses an integer system on a scale from 0 to 9 to measure the presence and partially measure the dose of the 4 GDMT classes.^5^ The OMT score has been clinically validated, and higher scores are associated with improved survival, reduced hospitalizations, and improvement in symptoms.^6,7^ The mOMT score is similar to the OMT score but accounts for a patient’s clinical eligibility for each of the 4 GDMT classes. The mOMT score has been clinically validated, with higher scores being associated with lower mortality and hospitalization rates.^8,9^ The KCMO score is more granular than the OMT and mOMT, as it considers the percentage of target dose achieved for the BB and ACEi/ARB/ARNI GDMT classes and considers therapy contraindications.^10^ Despite the KCMO score’s robustness and sensitivity in measuring the level of GDMT optimization, it has not yet been clinically validated. The MOS is similar to the KCMO score as it incorporates the percentage of target dose achieved for BB and ACEi/ARB therapies while considering eligibility.^11^ However, the available MOS computable algorithm is limited to user entry of MRA and select BB and ACEi/ARB therapies.

These scoring tools vary in their levels of nuance and may prove useful as metric tools for institutions in evaluating the success of their HF services. To date, however, they have largely been examined in isolation, limiting the ability to directly compare their relative performance. Evaluating these scores side by side in a single study population allows for a more comprehensive assessment of their predictive utility and may help identify which scoring method best aligns with clinically meaningful outcomes such as HF readmission.

## Methods

This was a retrospective, multi-center cohort study of patients hospitalized for new or existing HF and discharged between February 1, 2021, and July 31, 2023, from 5 large academic medical centers in California. The start date of February 1, 2021, was chosen based on the publication date of the 2021 Update to the 2017 ACC Heart Failure Guideline, when the SGLT2i class was first recommended by the American College of Cardiology.^12^ The index hospitalization was defined as the first hospitalization for a patient within the study period. The study was submitted to the Institutional Review Board at each participating center and deemed exempt. A data use agreement was established among the centers to ensure compliance with the Health Insurance Portability and Accountability Act, safeguarding human subjects’ data against unauthorized disclosure or use.

### Inclusion and Exclusion Criteria

Patients included in the study were at least 18 years of age with an ejection fraction ≤40% based on transthoracic echocardiogram (TTE), transesophageal echocardiogram (TEE), or cardiac magnetic resonance imaging (cMRI). Only patients discharged on at least one guideline-directed medical therapy (GDMT), including BB, ACEi, ARB, ARNI, MRA, or SGLT2i, were included to reduce the risk for residual confounding. To ensure adequate follow-up, patients were also required to have at least one subsequent healthcare encounter (e.g., any clinic visit, emergency department visit, or hospital readmission) within 90 days of discharge.

Patients were excluded if they died during the index hospitalization, were discharged to jail or prison, psychiatric institutions, hospice, left against medical advice, or transferred to an outside hospital. Additional exclusion criteria included acute coronary syndrome, stroke, coronary artery bypass grafting, valvular intervention, or cardiac arrest within 90 days before the index hospitalization, discharged on any intravenous (IV) inotrope, a history of restrictive cardiomyopathy (e.g., hypertrophic or amyloid), heart transplant, ventricular assist device, dialysis, or an estimated glomerular filtration rate (eGFR) <20 mL/min/1.73 m² as calculated by the Modification of Diet in Renal Disease equation.

### GDMT Scores

Medications and doses were collected from the discharge medication list on electronic medical records. The 4 GDMT classes and target doses were defined as BB, ACEi/ARB/ARNI, MRA, and SGLT2i in accordance with the AHA/ACC 2022 Heart Failure Guideline.^2^ In addition to counting the number of GDMT classes, medication regimens were transformed into the OMT score per the previously published framework (Supplemental Table S1).^5^ Doses of BB, ACEi, and ARBs were categorized by <50% or ≥50% maximal target dose and assigned up to 2 points. Three points were assigned for any dose of ARNI. The MRA and SGLT2i classes were assigned full points for any dose given a dose-response has not been clearly established for MRA, and SGLT2i were studied in HFrEF at 10 mg daily only.^13-16^ Point sums were then transformed into a fraction of total possible points (9 total) and multiplied by 100% to create a range from 0% (no points) to 100% (maximum possible points). The mOMT score used the same point allotment system as the OMT but additionally considered documented reasons for exclusion which were based on previously published studies (Supplemental Table S2).^8,17^ Patients with a reason for exclusion had the respective points excluded from the maximal total score, so that the mOMT denominator reflected only eligible medications. The KCMO score incorporated the 4 GDMT classes and was calculated first by dividing the total daily dose by target dose for BB and ACEi/ARB/ARNI.^10^ Target doses used in the calculations are presented in Supplemental Table S3. Second, the MRA drug class was assigned a total daily dose-to-target dose ratio of 1 for any dose or 0 for no treatment in accordance with the published methodology. Additionally, the SGLT2i class, which was not included in the original KCMO score, was assigned a ratio of 1 for any dose or 0 for no treatment. Lastly, the ratios were averaged across all eligible classes, and the resultant value was multiplied by 100% to create a range from 0% (no eligible GDMT used) to 100% (on target doses of all eligible GDMT). Patients with a reason for exclusion to a GDMT (Supplemental Table S2) had the drug class excluded from the score calculation. Example calculations for OMT, mOMT, and KCMO are shown in Supplemental Figure S1. The MOS score was not calculated in this study given its limited therapy selection in the computable algorithm.

### Outcomes

The outcomes of interest were the association of GDMT scores at the time of discharge on HF readmissions within 30 and 90 days of the index hospitalization. HF readmissions were identified through manual chart review of admission notes and defined as cases in which HF was listed as the primary or secondary reason for admission on the problem list.

### Data Collection

Patients were identified in the electronic medical record through a structured query language (SQL) database by querying for International Classification of Diseases Tenth Revision (ICD-10) codes for HF in the index hospitalization diagnosis (Supplemental Table S4).^18^ To avoid limitations associated with ICD-10 codes secondary to differences in user entry for database queries, other eligibility criteria, comorbidities, baseline characteristics, and readmission data were obtained and verified via manual chart review. Readmissions included hospitalizations at the 5 study institutions as well as external facilities identified through the Care Everywhere health information exchange feature in EPIC (EPIC Systems Corporation, Verona, WI). Only the first HF readmission up to 90 days post-discharge from the index hospitalization was collected. Data were securely stored on a REDCap server (Research Electronic Data Capture) hosted within one of the study centers. Upon completion of data collection, quality assurance was performed by identifying and querying out-of-range or unexpected values. These queries were addressed by the original data collectors through a review of the corresponding medical records, and any necessary corrections were made directly in the REDCap database.

### Statistical Analysis

Baseline characteristics were summarized using descriptive statistics including median, interquartile range (IQR), counts, or percentages, where appropriate. Continuous variables were analyzed by the Wilcoxon rank-sum test. Categorical variables were analyzed by chi-square test or Fisher exact test, as appropriate.

Mixed effect Cox proportional hazards models were used to estimate the association between the GDMT scores (i.e., GDMT count, OMT, mOMT, and KCMO) and 30- and 90-day hospital readmission. These models accounted for data clustering by incorporating random effects to account for variability across health systems.^19^ Hazard ratios (HR) for hospital readmission in the GDMT count and OMT scores were adjusted for race, age, sex, ejection fraction, new HF diagnoses, systolic blood pressure, heart rate, sodium, estimated glomerular filtration rate, chronic obstructive pulmonary disease history, cardiology post-discharge follow-up, and history of HF hospitalization. These covariates were selected a priori based on clinical judgment and evidence from the literature.^20^ The mOMT and KCMO models were adjusted for the same covariates as GDMT count and OMT, except systolic blood pressure, heart rate, and estimated glomerular filtration rate. These variables were excluded to avoid overadjustment since they are already embedded in the scores’ exclusion criteria.

Forest plots and Kaplan-Meier curves were used to depict the strength and direction of the association. Patients were censored at the time of new start of IV inotrope therapy, death, heart transplant, or LVAD implantation post-discharge.

An exploratory analysis was conducted to examine the relative contributions to the 90-day readmission outcome made by patients who met criteria for receiving 1 point in the BB and/or ACEi/ARB categories regardless of MRA or SGLT2i on the OMT score. Patients were divided into 2 groups: (1) minimal GDMT (i.e., individuals on 1% to <25% target BB dose and any ARNI dose, 1% to <25% target BB and ACEi/ARB dose, 1% to <25% target BB dose and no ACEi/ARB/ARNI, or 1% to <25% ACEi/ARB target dose and no BB) and (2) near-minimal GDMT (i.e., individuals on 25% to <50% target BB dose and any ARNI dose, 25% to <50% target BB dose and ACEi/ARB dose, 25% to <50% target BB and no ACEi/ARB/ARNI, or 25% to <50% ACEi/ARB target dose and no BB). The median OMT and percentage of patients with a HF readmission within 90 days were calculated for both categories.

All statistical tests were two-tailed, and a *P*-value <0.05 was considered statistically significant. All analyses were performed using R statistical software, version 4.3.2.

## Results

**Figure 1.**
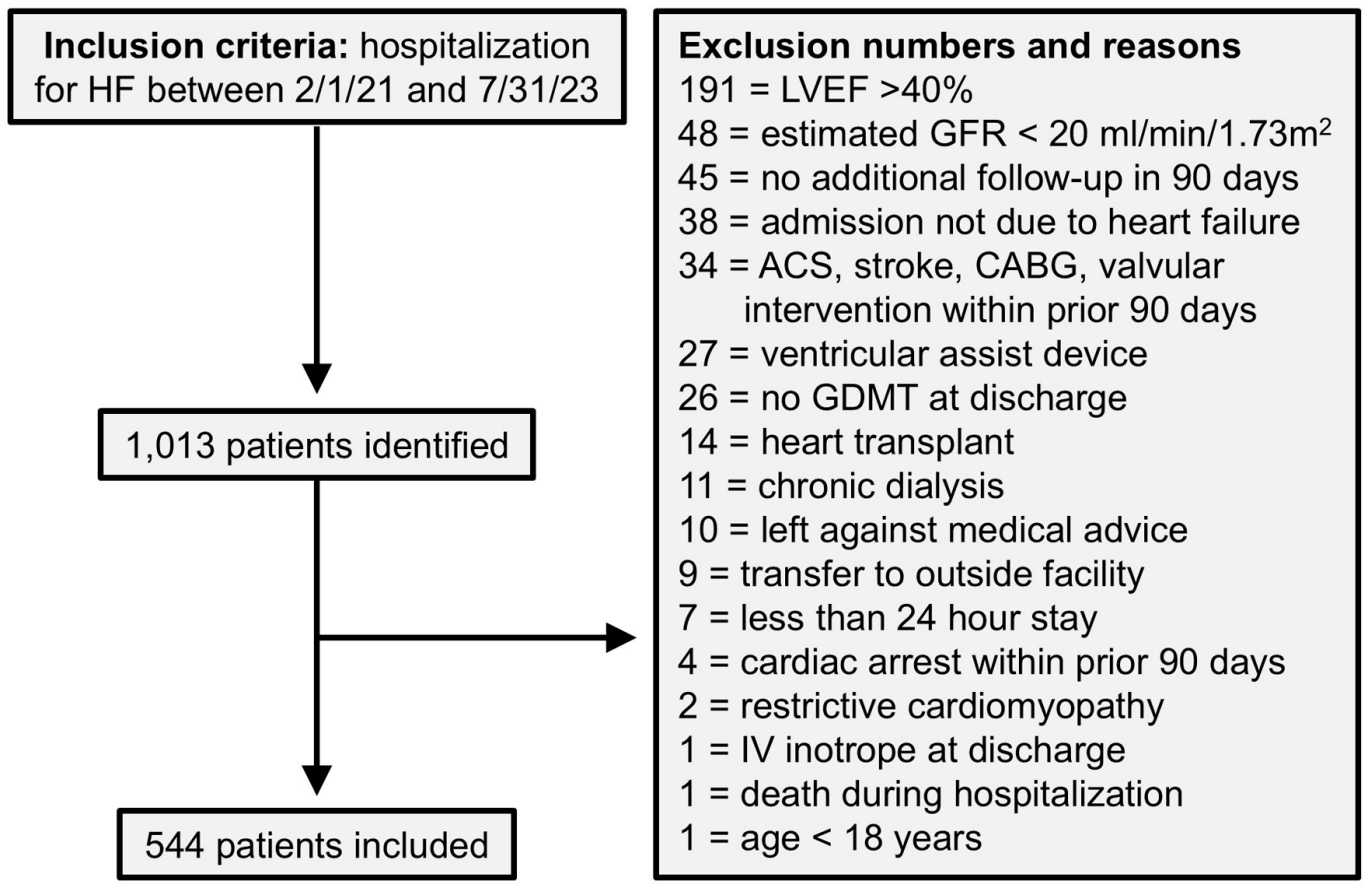
Study consort diagram. Abbreviations: ACS, acute coronary syndrome; CABG, coronary artery bypass graft; GDMT, guideline-directed medical therapy; GFR, glomerular filtration rate; HF, heart failure; IV, intravenous.

Between February 1, 2021, and July 31, 2023, a total of 1013 patients were screened across the five academic medical centers (Figure 1). Of these, 544 patients met the inclusion criteria. The overall population was a median 59 years of age, 23.7% female, and 39.3% white (Table 1). Most patients were discharged on ACEi/ARB/ARNI (77.9%) and beta blocker (87.3%), but utilization of MRA and SGLT2i was lower (59.9% and 50.0%, respectively). For GDMT count, 14.3% were on monotherapy, 27.0% were on dual therapy, 27.8% were on triple therapy, and 30.9% were on quadruple therapy. The overall median OMT, mOMT, and KCMO scores were 55.6% (IQR: 33.3-77.8), 66.7% (IQR: 44.4-86.5), and 37.5% (20.8-59.4), respectively. A total of 71 patients (13.1%) had a readmission within 30 days, and 146 (26.8%) had a readmission within 90 days.

**Table 1.** Baseline Characteristics.

Characteristic	Overall (n = 544)
Age, years (IQR)	59 (49-69)
Female, n (%)	129 (23.7)
Body-mass index, kg/m^2^ (IQR)	26.2 (22.6-31.3)
Ejection fraction, % (IQR)	24 (19-31)
Systolic blood pressure, mmHg (IQR)	109 (99-122)
Diastolic blood pressure, mmHg (IQR)	69 (62-80)
Heart rate, beats per minute (IQR)	82 (73-93)
Sodium, mEq/L (IQR)	137 (134-139)
Potassium, mEq/L (IQR)	4.2 (3.9-4.5)
Blood urea nitrogen, mg/dL (IQR)	26 (20-34)
Estimated GFR, mL/min/1.73^2^ (IQR)	68.4 (50.9-86.3)
Serum creatinine, mg/dL (IQR	1.19 (0.97-1.44)
Hemoglobin, g/dL (IQR)	13.2 (11.5-14.9)
Length of stay, days (IQR)	6 (4-9)
Race, n (%)	
White	214 (39.3)
Black	111 (20.4)
Asian	67 (12.3)
Other	50 (9.2)
Unknown	11 (2.0)
Native American	3 (0.6)
Hispanic/Latino ethnicity, n (%)	88 (16.2)
Medical history, n (%)	
New heart failure diagnosis	183 (33.6)
Atrial fibrillation/flutter	211 (38.8)
Coronary artery disease	173 (31.8)
Chronic obstructive pulmonary disease	76 (14.0)
Type 2 diabetes	174 (32.0)
Hypertension	290 (53.3)
Myocardial infarction	105 (19.3)
Stroke	53 (9.7)
Cardiac resynchronization therapy	45 (8.3)
Implantable cardioverter defibrillator	96 (17.6)
HF hospitalization within 12 months	107 (19.7)
30-day follow-up post-discharge, n (%)	
Cardiology	281 (51.7)
Medications, n (%)	
ACEi/ARB	216 (39.7)
ARNI	208 (38.2)
BB	475 (87.3)
MRA	326 (59.9)
SGLT2i	272 (50.0)
Loop diuretic	497 (91.4)
Digoxin	34 (6.3)
Hydralazine/nitrate	27 (5.0)
Ivabradine	7 (1.3)
Vericiguat	1 (0.2
GDMT scores	
Monotherapy, n (%)	78 (14.3)
Dual therapy, n (%)	147 (27.0)
Triple therapy, n (%)	151 (27.8)
Quadruple therapy, n (%)	168 (30.9)
OMT, % (IQR)	55.6 (33.3-77.8)
mOMT, % (IQR)	66.7 (44.4-86.5)
KCMO, % (IQR)	37.5 (20.8-59.4)

Values are medians, interquartile ranges (IQR), numbers, and percentages. Patients with and without a 30-day readmission are compared.

Abbreviations: ACEi, angiotensin-converting enzyme inhibitor; ARB, angiotensin receptor blocker; ARNI, angiotensin receptor-neprilysin inhibitor; BB, beta blocker; GDMT, guideline-directed medical therapy; GFR, glomerular filtration rate; HF, heart failure; KCMO, Kansas City Medical Optimization score; mOMT, modified optimal medical therapy score; MRA, mineralocorticoid receptor antagonist; OMT, optimal medical therapy score; SGLT2i, sodium-glucose cotransporter 2 inhibitor.

**Figure 2.**
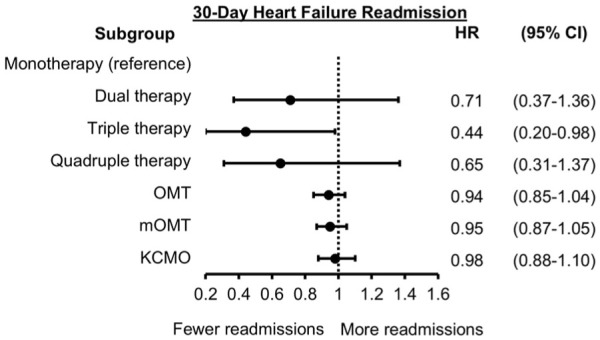
Forest plot for 30-day HF readmission. Adjusted hazard ratios (HR) and 95% confidence intervals (CI) are shown. Monotherapy is reference for dual, triple and quadruple therapy. The OMT, mOMT, and KCMO variables are modeled per 10 percentage point increase. Abbreviations: KCMO, Kansas City Medical Optimization score; mOMT, modified optimal medical therapy score; OMT, optimal medical therapy score.

As shown in Figure 2, only triple therapy GDMT count was associated with a lower hazard of 30-day readmission as compared to monotherapy (HR: 0.44, 95% CI: 0.20-0.98). The other GDMT count groups, such as dual therapy (HR: 0.71, 95% CI: 0.37-1.36) and quadruple therapy (HR: 0.65, 95% CI: 0.31-1.37), did not show statistical significance. Similarly, OMT (HR: 0.94, 95% CI: 0.85-1.04), mOMT (HR: 0.95, 95% CI: 0.87-1.05), and KCMO (HR 0.98, 95% CI: 0.88-1.10) were not associated with 30-day readmission.

**Figure 3.**
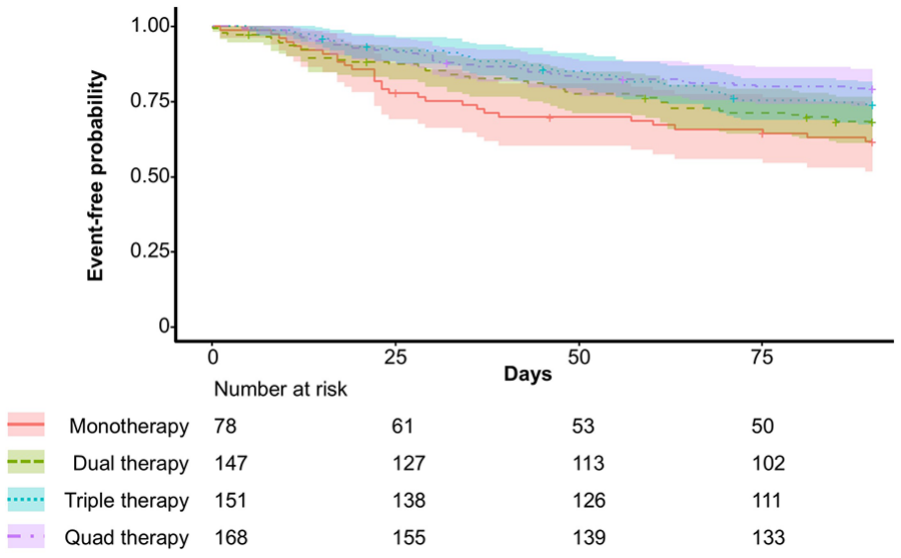
Kaplan-Meier curves for time-to-HF readmission within 90 days. Data is unadjusted. Patients were censored at the time of new start of intravenous inotrope therapy, death, heart transplant, or left-ventricular assist device implantation post-discharge which are indicated by crosses.

**Figure 4.**
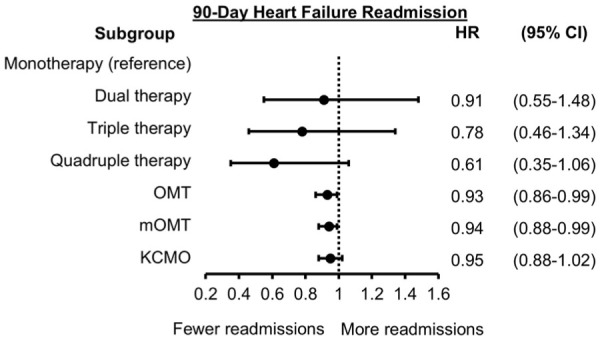
Forest plot for 90-day HF readmission. Adjusted hazard ratios (HR) and 95% confidence intervals (CI) are shown. Monotherapy is reference for dual, triple and quadruple therapy. The OMT, mOMT, and KCMO variables are modeled per 10 percentage point increase. Abbreviations: KCMO, Kansas City Medical Optimization score; mOMT, modified optimal medical therapy score; OMT, optimal medical therapy score.

The probability for 90-day HF readmission by GDMT count is depicted in Figure 3. The GDMT count scores for dual (HR: 0.91, 95% CI: 0.55-1.48), triple (HR: 0.78, 95% CI: 0.46-1.34), and quadruple therapy (HR: 0.61, 95% CI: 0.35-1.06) did not reach statistical significance, despite showing a trend toward benefit with increasing the number of agents at discharge (Figure 4). Higher OMT (HR: 0.93, 95% CI: 0.86-0.99) and mOMT (HR: 0.94, 95% CI: 0.88-0.99) scores were associated with a statistically significant reduction in risk in HF readmission. The KCMO score (HR: 0.95; 95% CI: 0.88-1.02) was not significantly associated with reduction in risk for HF readmission at 90 days.

In the exploratory analysis, 156 patients (28.7%) received minimal GDMT, while 103 (18.9%) received near-minimal GDMT. The median OMT score for both groups was 33.33 (IQR: 11.11-55.56). Within 90 days, 61 patients in the minimal GDMT group and 39 in the near-minimal GDMT group experienced an HF readmission. Although patients receiving minimal GDMT comprised only 28.7% of the total study population, they accounted for 39.4% of all 90-day readmissions (n = 155). In contrast, those receiving near-minimal GDMT represented 18.9% of the cohort but contributed 25.2% of all readmissions.

## Discussion

In this analysis of 544 patients hospitalized for HF across 5 large academic medical centers, we estimated the association of GDMT count, OMT, mOMT, and KCMO scores with 30- and 90-day HF readmissions. None of the 4 scores were significantly associated with 30-day HF readmissions. Only OMT and mOMT were predictive of a readmission at 90 days. These findings highlight a possible discordance among the available scoring systems, necessitating further validation of their clinical utility.

Our finding that OMT and mOMT were associated with HF readmissions while KCMO was not could be explained by the relative sensitivity of the scoring systems. Due to their integer-based point assignment, the OMT and mOMT could result in inflated scores for patients with suboptimal therapy.^10^ For example, metoprolol succinate 100 mg daily is allotted the same number of points as 200 mg daily (2 points). Similarly, metoprolol succinate 12.5 mg daily is allotted the same number of points as 50 mg daily (1 point). This lack of complete dose differentiation is important to consider given the BB and ACEi/ARB/ARNI classes are known to have a dose-responsive relationship with HF outcomes.^21,22^ In our exploratory analysis, minimal GDMT regimens (i.e., 1 to <25% target BB and/or ACEi/ARB doses) and near-minimal GDMT regimens (i.e., 25 to <50% target BB and/or ACEi/ARB doses) had equivalent OMT scores. However, patients on minimal GDMT had a greater contribution to total 90-day HF readmissions than those on near-minimal GDMT. Together, these findings suggest that minimal GDMT doses could have biased low OMT scores toward even greater risk for readmission, leading to an increased likelihood of the score predicting readmissions. The mOMT score, while it excludes ineligible GDMT, would likely be biased in the same way given its integer-based allotment of points like OMT.

The currently published GDMT scores could be refined. The scores assume equal or near-equal weighting of each GDMT component, applying an additive framework that may oversimplify the true pharmacodynamic effects of various therapy combinations. Future iterations could benefit from differential weighting based on class-specific efficacy and clinical impact. Dose intensity should also be captured, particularly for classes with well-established dose-response relationships.^21,22^ Researchers could also incorporate a consideration of intolerance to higher doses of medications to better quantify GDMT in sicker populations, which has been piloted in another study.^23^ Current scoring models only capture treatment at a single timepoint, which may fail to reflect the cumulative impact of sustained therapy. Thus, incorporating duration of GDMT exposure as described through methods in the literature may enhance the accuracy of scoring systems.^24^ While these enhancements could improve the validity and predictive value of GDMT scores, they must be carefully balanced against the need for simplicity to ensure feasibility and adoption in clinical practice.

Despite any biases and need for improvement, GDMT scores still have real-world utility. A substantial body of evidence supports that greater use of more GDMT, both in terms of class and dose, is strongly associated with improved morbidity and mortality.^4,25^ Thus, any metric that identifies patients with potential for optimization is clinically meaningful. Two recent studies have explored the integration of GDMT count into the electronic health record to promote optimization in both outpatient and inpatient settings.^26,27^ Both studies identified patients not on quadruple therapy and utilized embedded best practice alerts in the electronic health record to recommend changes to GDMT. The outpatient study showed significant improvements in overall GDMT after 30 days, while the inpatient study did not. Reasons for not finding significance were thought secondary to potential alert fatigue in the hospital setting, pressure to limit length of stay, diffusion of responsibility, and expectation that GDMT optimization is intended for the outpatient setting. While these studies showed mixed results and a need for refinement in methods for utilizing GDMT scores, they overall demonstrate the potential of scoring systems in promoting therapy optimization.

This study has limitations. First, the study population was relatively small, and findings may not extend to all centers across the United States given patient data was extracted from institutions localized to the West Coast. Second, Care Everywhere is restricted to participating institutions and, thus, our chart review may not have captured all possible readmissions for HF or deaths at all outside hospitals. Third, our readmission rate (13.1% for 30-day readmission) is lower than the reported national average in the Hospital Readmissions Reduction Program under the Centers for Medicare and Medicaid Services (CMS) (~20%).^28^ This difference is attributable to our study design, which included only the first hospitalization in the study period and its first readmission up to 90 days. In contrast, CMS calculates rates using a rolling 30-day window anchored to each qualifying discharge, capturing every eligible HF hospitalization and its readmissions.^29^ This methodological difference limits the generalizability of our findings to national benchmarks. Fourth, an inherent limitation to all scores used in this study is their vulnerability to non-adherence and post-discharge changes in GDMT, which may have introduced bias in assessing the true benefit of GDMT on readmissions. Fifth, the findings for GDMT count (e.g. triple therapy at 30 days) should be interpreted with caution given the wide confidence intervals and limited sample size. Sixth, we did not model competing events (e.g. death, IV inotrope, LVAD placement, and heart transplantation) which could result in overestimation of GDMT impact on readmission. However, the percentage of patients with a competing event up to 90 days was low (4%), suggesting that our observed associations may not be solely driven by survivorship bias.^30^ Lastly, GDMT eligibility was based on statements by clinicians in patient notes, though they were confirmed with retrospective review for objective data (e.g. potassium and serum creatinine).

## Conclusion and Relevance

In this multi-center retrospective cohort study, we found that higher OMT and mOMT scores at hospital discharge were significantly associated with reduced 90-day HF readmissions, whereas GDMT count and KCMO scores were not. These findings underscore the potential utility of simpler scoring systems that incorporate class presence and eligibility but also highlight important limitations in current frameworks, including their inability to fully differentiate dose intensity and sustained use. Future scoring systems may benefit from greater granularity, class-specific weighting, and longitudinal treatment assessment. Despite these limitations, GDMT scores remain valuable tools to identify patients with optimization opportunities and may aid in the development of targeted interventions to improve HF outcomes. Future prospective studies are warranted to validate and refine these scoring systems for broader clinical implementation.

## Supplemental Material

sj-docx-1-aop-10.1177_10600280251387249 – Supplemental material for Assessment of Guideline-Directed Medical Therapy Optimization Scores and Readmission Risk in Heart Failure With Reduced Ejection Fraction Supplemental material, sj-docx-1-aop-10.1177_10600280251387249 for Assessment of Guideline-Directed Medical Therapy Optimization Scores and Readmission Risk in Heart Failure With Reduced Ejection Fraction by Hanna Jensen, Max Staskauskas, Sara Strome, Jethro Pobre, Nghi Le, Cameryn Nakamura, Mina Fahmy, Benjamin Dang, Yvette Hellier, Jaekyu Shin, Tiffany K. Pon, Trina Huynh, Jennifer Namba, Louise T. Wang, Christine Cadiz, Zaid Yousif and Andrew Willeford in Annals of Pharmacotherapy
