# Supplementary material for: Assessment of Guideline-Directed Medical Therapy Optimization Scores and Readmission Risk in Heart Failure With Reduced Ejection Fraction
Source: Ann Pharmacother. 2025 Nov 14;60(6):546–54. doi: 10.1177/10600280251387249 (PMC13172379; doi:10.1177/10600280251387249)
Supplement: sj-docx-1-aop-10.1177_10600280251387249 – Supplemental material for Assessment of Guideline-Directed Medical Therapy Optimization Scores and Readmission Risk in Heart Failure With Reduced Ejection Fraction [file sj-docx-1-aop-10.1177_10600280251387249.docx]

| **Table S1. Optimal Medication Therapy Score** | | |
| --- | --- | --- |
| Therapy | Dose^#^ | Points |
| BB | None | 0 |
|  | <50% target | 1 |
|  | ≥50% target | 2 |
| ACEi/ARB/ARNI | None | 0 |
|  | <50% target ACEi/ARB | 1 |
|  | ≥50% target ACEi/ARB | 2 |
|  | ARNI any dose | 3 |
| MRA | None | 0 |
|  | Any dose | 2 |
| SGLT2i | None | 0 |
|  | Any dose | 2 |
| Abbreviations: ACEi, angiotensin II converting enzyme inhibitor; ARB, angiotensin II receptor blocker; ARNI, angiotensin receptor II blocker/neprilysin inhibitor; BB, beta blocker; MRA, mineralocorticoid receptor antagonist; SGLT2i, sodium/glucose cotransporter-2 inhibitor.  ^#^Doses are defined according to the 2022 AHA/ACC HFSA Heart Failure guideline | | |

| **Table S2. Reasons to exclude used in mOMT and KCMO calculations** | |
| --- | --- |
| Therapy | Reasons to exclude |
| BB | Heart rate <60, symptoms of hypotension on lowest dose* |
| ACEi/ARB/ARNI | Potassium ≥5, angioedema to ACEi/ARB/ARNI^†^, symptoms of hypotension on lowest dose*, acute kidney injury^‡^ |
| MRA | Potassium ≥5, eGFR <30 at baseline, acute kidney injury^‡^ |
| SGLT2i | eGFR <20 at baseline, recurrent urinary tract infection |
| Abbreviations: ACEi, angiotensin II converting enzyme inhibitor; ARB, angiotensin II receptor blocker; ARNI, angiotensin receptor II blocker/neprilysin inhibitor; BB, beta blocker; eGFR, estimated glomerular filtration rate; MRA, mineralocorticoid receptor antagonist; SGLT2i, sodium/glucose cotransporter-2 inhibitor.  *Symptoms of hypotension were defined as dizziness, lightheadedness, or syncope documented in the provider note as attributable to the therapy  ^†^ARB is included if angioedema to ACEi only  ^‡^Acute kidney injury defined as an increase in serum creatinine by ≥1.5 times baseline | |

| **Table S3. Target Doses for Guideline-Directed Medical Therapy** | | | |
| --- | --- | --- | --- |
| Class | Drug | Target dose (mg) | Dose frequency |
| BB | Bisoprolol | 10 | Daily |
|  | Carvedilol | 25 | Twice daily |
|  | Metoprolol succinate | 200 | Daily |
| ACEi | Benazepril^†^ | 20 | Daily |
|  | Enalapril | 10 | Twice daily |
|  | Lisinopril | 20 | Daily |
|  | Ramipril | 10 | Daily |
| ARB | Losartan | 50 | Daily |
|  | Valsartan | 160 | Twice daily |
| ARNI | Sacubitril/valsartan | 97/103 | Twice daily |
| Listed therapies reflect those represented in the study data.  Abbreviations: ACEi, angiotensin II converting enzyme inhibitor; ARB, angiotensin II receptor blocker; ARNI, angiotensin receptor II blocker/neprilysin inhibitor; BB, beta blocker.  ^†^The target dose of benazepril in heart failure with reduced ejection fraction has not been established. This study identified 12 patients on benazepril and used a target dose of 20 mg daily based on methodology used in Ribner HS, Sagar KB, and Glasser SP et al. *J Clin* *Pharmacol*.1990;30(12):1106-1111. | | | |

| Table S4. ICD-10 Codes Used to Identify Diagnoses | |
| --- | --- |
| Diagnoses | ICD-10 Code |
| Atrial fibrillation/flutter | I48* |
| Acute coronary syndrome | I24.9 |
| Coronary artery disease | I25* |
| Chronic kidney disease | N18* |
| Diabetes | E10*, E11*, E13*, E08*, O24* |
| Hyperlipidemia | E78* |
| Hypertension | I10 |
| Stroke | I63* |
| Heart failure | I50* |
| Amyloidosis | E85.4 |
| Hypertrophic cardiomyopathy | I42.1, I42.2 |
| Congenital heart disease | Q24.9 |
| Ventricular assist device | Z95.811 |

**Figure S1. Example Optimal Medical Therapy (OMT), Modified Optimal Medical Therapy (mOMT), and Kansas City Medical Optimization (KCMO) Score Calculations.**

Patient A is taking metoprolol succinate 100 mg daily, valsartan 160 mg twice daily, and dapagliflozin 10 mg daily. The patient has a history of hyperkalemia while on spironolactone.

$$OMT score= \frac{Sum of points}{Total possible points} x 100\%$$

$$OMT score= \frac{2 \left( \geq50\% target dose BB \right)+2 \left( \geq50\% target dose ARB \right)+2 (any dose SGLT2i)}{9} x 100\%= \frac{6}{9} x 100\%=66.7\%$$

$$mOMT score= \frac{Sum of points}{Total possible points (adjusted for intolerance)} x 100\%$$

$$mOMT score= \frac{2 \left( \geq50\% target dose BB \right)+2 \left( \geq50\% target dose ARB \right)+2 (any dose SGLT2i)}{7} x 100\%= \frac{6}{7} x 100\%=85.7\%$$

$$KCMO score= \frac{\Sigma(dose to target ratios for each eligible class)}{Number of elgible classes} x 100\%$$

$$KCMO score= \frac{100/200 \left( \mathrm{metoprolol} \right)+320/320 \left( \mathrm{valsartan} \right)+1 (dapagliflozin)}{3} x 100\%= \frac{2.5}{3} x 100\%=83.3\%$$
